# Assessment of long-range cross-correlations in cardiorespiratory and cardiovascular interactions

**DOI:** 10.1098/rsta.2020.0249

**Published:** 2021-12-13

**Authors:** Akio Nakata, Miki Kaneko, Chinami Taki, Naoko Evans, Taiki Shigematsu, Tetsuya Kimura, Ken Kiyono

**Affiliations:** ^1^ Graduate School of Engineering Science, Osaka University, Osaka 560-8531, Japan; ^2^ Development Department, Union Tool Co., Tokyo 140-0013, Japan; ^3^ Division of Physical and Health Education, Setsunan University, Osaka 572-8508, Japan; ^4^ Graduate School of Human Development and Environment, Kobe University, Kobe 657-8501, Japan

**Keywords:** long memory process, complex system interaction, cross-spectral analysis

## Abstract

We propose higher-order detrending moving-average cross-correlation analysis (DMCA) to assess the long-range cross-correlations in cardiorespiratory and cardiovascular interactions. Although the original (zeroth-order) DMCA employs a simple moving-average detrending filter to remove non-stationary trends embedded in the observed time series, our approach incorporates a Savitzky–Golay filter as a higher-order detrending method. Because the non-stationary trends can adversely affect the long-range correlation assessment, the higher-order detrending serves to improve accuracy. To achieve a more reliable characterization of the long-range cross-correlations, we demonstrate the importance of the following steps: correcting the time scale, confirming the consistency of different order DMCAs, and estimating the time lag between time series. We applied this methodological framework to cardiorespiratory and cardiovascular time series analysis. In the cardiorespiratory interaction, respiratory and heart rate variability (HRV) showed long-range auto-correlations; however, no factor was shared between them. In the cardiovascular interaction, beat-to-beat systolic blood pressure and HRV showed long-range auto-correlations and shared a common long-range, cross-correlated factor.

This article is part of the theme issue ‘Advanced computation in cardiovascular physiology: new challenges and opportunities’.

## 1. Introduction

Heart rate variability (HRV) in a healthy individual displays complex fluctuations even during a resting state [1,2]. One of the typical characteristics of HRV is $1/f^{\beta }$-type scaling in the power spectrum, also known as the 1/f fluctuation [3]. The scaling exponent β during usual daytime activities is controlled to one in healthy young adults [4]. Alterations in β are associated with ageing, disease and mortality [2,5,6]. Characterizing and understanding HRV fluctuations are essential in biomedical applications.

The 1/f fluctuation means that the observed time series has a non-trivial long-range auto-correlation. Specifically, the auto-correlation function of the time series (or its increment) exhibits a slow decay that follows a power law [7] (or logarithmic decay when β=1 [8]). Long-range correlations are hallmarks of complex systems, including living organisms [9–11]. To date, several mathematical models have been proposed to generate the 1/f fluctuation time series [12,13]. However, the underlying mechanisms of fluctuations in biological systems remain unclear.

The biomedical significance of HRV has been well-established [1,2]. For instance, as a non-invasive tool for assessment of autonomic nervous system function, frequency-domain indices, high-frequency (HF: 0.15–0.40 Hz) and low-frequency (LF: 0.04–0.15 Hz) band powers, have been widely used. The HF power in the frequency band around 0.25 Hz is primarily associated with the synchronization between HRV and respiration cycle, mediated by vagal parasympathetic activity. Therefore, HF power has been used as a measure of cardiac parasympathetic activity. The LF power in the frequency band around 0.1 Hz is associated with the synchronization between HRV and arterial blood pressure (BP) oscillation known as the Mayer wave. Although the LF component is believed to be modulated by both sympathetic and parasympathetic nerve activities, its association with sympathetic nerve activity is still under debate [14]. To date, the effects of respiration and BP control on HRV have been evaluated through frequency-specific components such as the HF and LF powers [15–19]. By contrast, the relation between 1/f fluctuations and respiratory-cardiovascular interactions has not been investigated. Therefore, we explore this relation to gain new insights into the complex dynamics of HRV.

Recently, we developed higher-order detrending moving-average cross-correlation analysis (DMCA) to detect the existence of a common long-range correlated factor hidden in two different processes [20]. In our method, non-stationary trends embedded in each time series are removed using a Savitzky–Golay detrending filter [21], because such trends adversely affect the scaling exponent estimation [22]. We have established a rigorous mathematical foundation of the higher-order DMCA [20]. In this paper, we use this method to evaluate cardiorespiratory and cardiovascular interactions. We analyse heart rate (HR) and respiration variability to study cardiorespiratory interactions and HR and beat-to-beat BP variability to study cardiovascular interactions.

## 2. Long-range cross-correlation

In this section, we briefly illustrate the long-range cross-correlations in bivariate stochastic processes [23] and its characterization using DMCA [20]. The long-range cross-correlation between two different processes implies that the both processes are affected by a common noise or have a common long-range correlated factor. Therefore, the detection of long-range cross-correlations provides insight into how two processes interact.

### (a) Basics of long-range cross-correlation

The basic properties of long-range cross-correlated processes are described under an assumption of second-order stationarity. For a sake of simplicity, let us consider a bivariate stochastic process $\left\{(X^{\left(1\right)}[i],X^{\left(2\right)}[i])\right\}$ at integer time steps. In this process, we assume that the cross-correlation function between $X^{\left(1\right)}[i]$ and $X^{\left(2\right)}[i]$ is given by a power-law decay around the time lag $\kappa_{0}$

2.1 $$C_{12}(k)=E\;[X^{\left(1\right)}[i-\kappa_{0}]\;X^{\left(2\right)}[i-k]]∼|k-\kappa_{0}{|}^{-\gamma }\;(|k-\kappa_{0}|\gg 1),$$

where the scaling exponent γ is defined in the range of 0<γ<1, $\kappa_{0}$ is a constant, E[ ⋅ ] denotes the expectation over ensembles, and the tilde symbol denotes proportionality. In this paper, we denote random variables by capital letters and their realizations by the corresponding lowercase letters. The power-law decay of the cross-correlation means the nonzero correlation persists over a long range of time lag k. This persistent cross-correlation can be characterized by γ, while the power-law decay of the correlation has no characteristic time scale.

The cross-correlation can also be characterized by the cross-power spectral density, because this spectral density is given by the discrete Fourier transform of the cross-correlation function [24]. Under the assumption of equation (2.1), the corresponding cross-power spectral density is given by

2.2 $$|S_{12}(f)|∼f^{-\beta }\;(|f|\ll 1/2),$$

where f is the frequency, and the asymptotic scaling relation β=1−γ holds for 0<β<1 [25]. Although this scaling relation holds only for 0<γ<1 due to the stationarity condition, the range of the scaling exponent β is not restricted to this range. Therefore, a scaling analysis using the cross-power spectral density can be applied to diffusive non-stationary processes such as bivariate fractional Brownian motions [26].

However, a spurious scaling exponent can emerge from non-stationary smooth trends embedded in time series [20]. Therefore, detrending-operation-based approaches, such as detrended cross-correlation analysis (DCCA) [27] and DMCA [20,28], have been introduced to achieve reliable estimations of the scaling exponent. DCCA is one of the most widely used methods to detect long-range cross-correlations in real-world time series. However, DMCA has clear advantages over DCCA. For example, DMCA is mathematically simple and has a well-established formulation (see below for details); it also performs detrending with a linear frequency response [29] and a fast implementation algorithm [30]. Consequently, we employ DMCA in our analysis.

### (b) Detrending moving-average cross-correlation analysis

The mathematical foundation of DMCA is established using the framework of a generalized cross random walk analysis (CRWA) [20]. In this framework, we consider a bivariate time series $\left\{(X^{\left(1\right)}[i],X^{\left(2\right)}[i])\right\}$ and its mean cross-increment defined as

2.3 $$F_{12}^{2}(s,\kappa )=E\;[\Delta_{s}Y^{\left(1\right)}[i-\kappa ]\Delta_{s}Y^{\left(2\right)}[i]],$$

where $\Delta_{s}Y^{\left(l\right)}[i]$ is defined as a weighted partial sum with ${\left\{w_{s}(j)\right\}}_{j=1}^{s}$,

2.4 $$\Delta_{s}Y^{\left(l\right)}[i]=\sum_{j=1}^{s}w_{s}(j)\;X^{\left(l\right)}[i+j]\;(l=1,2).$$

The weights $\left\{w_{s}(j)\right\}$ are assumed to satisfy the self-affinity condition (at least asymptotically)

2.5 $$w_{s}(k)=w_{rs}(rk).$$

The simplest choice that fulfils this condition is $w_{s}(k)=1$ (1≤k≤s).

Under the assumption of second-order stationarity, the relations among $F_{12}(s,\kappa )$, $C_{12}(k)$ and $|S_{12}(f)|$ is given by

2.6 $$F_{12}^{2}(s,\kappa )=\sum_{k=-s}^{s}C_{12}(k+\kappa )\;L(k,s)=\int_{-1/2}^{1/2}\;|S_{12}(f)||G_{s}(f){|}^{2}\;\text{d}f,$$

where L(k,s) is defined as

2.7 $$L(k,s)=\sum_{j=1}^{s-|k|}w_{s}(j)\;w_{s}(j+|k|),$$

and $G_{s}(f)$ is defined as

2.8 $$G_{s}(f)=\sum_{k=1}^{s}w_{s}(k)\;{\text{e}}^{-i2\pi fk},$$

where i denotes the imaginary unit. Moreover, the relation between $|G_{s}(f){|}^{2}$ and L(k,s) is given by

2.9 $$|G_{s}(f){|}^{2}=\sum_{k=-s}^{s}L(k,s)\;\cos(2\pi fk).$$

This approach provides another scaling analysis as follows:

2.10 $$F_{12}(s,\kappa )\equiv \sqrt{|F_{12}^{2}(s,\kappa )|}∼s^{\alpha },$$

where α is the scaling exponent. Note that if $X^{\left(1\right)}$ and $X^{\left(2\right)}$ are negatively correlated, then $F_{12}^{2}(s,\kappa )$ takes a negative value.

When $X^{\left(1\right)}[i]$ and $X^{\left(2\right)}[i]$ do not have a long-range correlation with each other, $F_{12}^{2}(s,\kappa )$ drifts through positive and negative values, and the scaling analysis of $F_{12}(s,\kappa )$ (equation (2.10)) does not work. Therefore, to check for uncorrelated cases, we evaluate the multi-scale cross-correlation coefficient [31] as follows:

2.11 $$\rho_{12}(s,\kappa )=\frac{F_{12}^{2}(s,\kappa )}{F_{1}(s)\;F_{2}(s)},$$

where $F_{l}(s)$ (l=1,2) is

2.12 $$F_{l}(s)=E\;[{\left(\Delta_{s}Y^{\left(l\right)}[i]\right)}^{2}]\;(l=1,2).$$

When $C_{12}(k)=0$, $E\;[F_{12}(s,\kappa )]=0$, and $\rho_{12}(s,\kappa )=0$.

The choice of the weights ${\left\{w_{s}(k)\right\}}_{k=1}^{s}$ can improve the performance of the scaling analysis. An optimal choice of $\left\{w_{s}(k)\right\}$ is derived from higher-order DMCA. The DMCA procedure is summarized as follows: (1) The observed bivariate time series ${\left\{(x^{\left(1\right)}[i],x^{\left(2\right)}[i])\right\}}_{i=1}^{N}$ with zero means and finite variances are integrated separately. (2) The smoothly varying trends ${\tilde{y}}_{s}^{\left(l\right)}[i]$ included in each of $y^{\left(l\right)}[i]$ (l=1,2) are approximated by a Savitzky–Golay smoothing filter and removed from the integrated time series. (3) The root-mean cross-increment at each scale s is estimated by the detrended profile $y^{\left(l\right)}[i]-{\tilde{y}}_{s}^{\left(l\right)}[i]$. The higher-order DMCA employs a Savitzky–Golay smoothing filter to remove non-stationary trends more effectively than the original (zeroth-order) DMCA, which uses a simple moving-average filter. The filtered time series are obtained by the values of the least-squares polynomial at the centre point of each sub-interval with a window length s. The Savitzky–Golay filter has two parameters: the order of the least-squares polynomial m and the sub-interval length s, where m and s are restricted to even and odd numbers, respectively. Note that the zeroth-order Savitzky–Golay filter is equivalent to the simple moving-average filter. Using the Savitzky–Golay smoothing filter with an even polynomial order m, an arbitrary polynomial curve with degree (m+1) can be traced completely. Thus, DMCA using a higher-order Savitzky–Golay filter can well eliminate non-stationary trends locally approximated by a higher degree polynomial.

In the higher-order DMCA, the weight in equation (2.4) is given by

2.13 $$w_{s}(k)=\left\{ \begin{array}{ll} \Theta \left(\frac{s+1}{2}-k\right)-\sum_{j=0}^{m}b_{1,j+1}\sum_{r=k}^{s}{\left(r-\frac{s+1}{2}\right)}^{j} & \text{for}\;1\leq k\leq s\\ 0 & \text{for otherwise} \end{array}\right.,$$

where Θ(x) is the unit step function, which equals 0 for x<0 and 1 for x≥0, and $b_{i,j}(s)$ are elements of the inverse matrix of

2.14 $$B_{m}(s)=\sum_{j=-(s-1)/2}^{(s-1)/2}\left[\begin{array}{ccccc} 1 & 0 & j^{2} & \cdots & j^{m}\\ 0 & j^{2} & 0 & \cdots & 0\\ j^{2} & 0 & j^{4} & \cdots & j^{m+2}\\ \vdots & \vdots & \vdots & \ddots & \vdots \\ j^{m} & 0 & j^{m+2} & \cdots & j^{2m} \end{array}\right].$$

For a practical application of DMCA that analyses an observed bivariate time series $\left\{(x^{\left(1\right)}[i],x^{\left(2\right)}[i])\right\}$, we calculate

2.15 $$F_{12}(s,\kappa )=\sqrt{{\left\langle \Delta_{s}y^{\left(1\right)}[i-\kappa ]\Delta_{s}y^{\left(2\right)}[i]\right\rangle }_{i}},$$

where $\Delta_{s}y^{\left(l\right)}[i]=\sum_{j=1}^{s}w_{s}(j)\;x^{\left(l\right)}[i+j]$ (l=1,2). In this estimation, we employ the temporal sample mean operator defined as

2.16 $${\left\langle x[i]\right\rangle }_{i}=\frac{1}{N}\sum_{i}x[i],$$

where i is the temporal sequence number, and N is the total number of samples. Finally, the slope of the plot of $\log F_{12}(s,\kappa )$ versus log⁡s provides an estimate of α.

The methodological properties of DMCA are summarized in table 1. The detrendable polynomial degree is the upper limit of the polynomial degree of the trends embedded in the observed time series before integration. Analytical forms of kernels L(k,s) and $|G_{s}(f){|}^{2}$ were shown in [20]. In addition, when $\kappa =\kappa_{0}$, we can derive the scaling relations analytically as

2.17 $$\alpha =1-\gamma /2\;\left(\frac{1}{2}<\alpha <1\right)$$

and

2.18 $$\alpha =\frac{\beta +1}{2}\;(0<\alpha <2m+1).$$

Because the relations listed above require that $\kappa =\kappa_{0}$, an estimation of the time lag $\kappa_{0}$ is required prior to performing a DMCA. Note that a lag estimation is always required for the time-domain analyses of long-range cross-correlations, such as the cross-correlation function and DCCA.

**Table 1.** Properties of mth-order DMCA.

m	detrendable polynomial degree	detectable range of α	corrected time scale $\tilde{s}$
0	0	0<α<2	s/1.00
2	2	0<α<4	s/1.93
4	4	0<α<6	s/2.74

To determine the time lag κ, we estimate the detrended cross-covariance of the observed bivariate time series ${\left\{(x^{\left(1\right)}[i],x^{\left(2\right)}[i])\right\}}_{i=1}^{N}$ as

2.19 $$K_{12}(k)={\left\langle (x^{\left(1\right)}[i]-{\tilde{x}}_{s}^{\left(1\right)}[i])(x^{\left(2\right)}[i+k]-{\tilde{x}}_{s}^{\left(2\right)}[i+k])\right\rangle }_{i},$$

where ${\tilde{x}}_{s}^{\left(l\right)}[i]$ (l=1,2) is a time series filtered by a Savitzky–Golay filter with a window length of s, and s is a value within the scaling range. The choice of an optimal lag k is determined by the maximum value of $|K_{12}(k)|$ (equation (2.19)).

In the conventional DMCA (and in DCCA), the reciprocal relation s≈1/f does not always hold. Therefore, a correction must be applied to the scale s to confirm the consistency of the detected characteristic scale in the domains of time and frequency. As we pointed out in [20], the time-frequency relation is distorted because of the band-pass filtering property of the detrending procedure in DMCA. Consequently, we use a corrected time scale $\tilde{s}$ (table 1). For instance, $\tilde{s}\approx 1/f$ holds approximately when the corrected scale $\tilde{s}=s/1.93$ is used for the second-order DMCA.

## 3. Analysis methods and data

### (a) Analysis methods

Here, we briefly summarize the methodology for practical applications of long-range cross-correlation analysis using DMCA.

#### (i) Long-range auto- and cross-correlation analysis

We first assessed the long-range auto-correlation of each univariate time series. We employed the second-order detrending moving average analysis (DMA) [22], which is the univariate version of the second-order DMCA. In DMA, we consider an estimator of the root-mean square deviation for the observed time series {x[i]} defined as

3.1 $$F(s)=\sqrt{{\left\langle \Delta_{s}y{\left[i\right]}^{2}\right\rangle }_{i}},$$

where $\Delta_{s}y[i]$ is defined as $\Delta_{s}y[i]=\sum_{j=1}^{s}w_{s}(j)\;x[i+j]$, and the weights $\left\{w_{s}(j)\right\}$ are given by equation (2.13). Under the assumption of second-order stationarity, the relation among F(s), the auto-correlation C(k) and the power spectrum S(f) is given by

3.2 $$F^{2}(s)=\sum_{k=-s}^{s}C(k)\;L(k,s)=\int_{-1/2}^{1/2}\;S(f)|G_{s}(f){|}^{2}\;\text{d}f.$$

Note that L(k,s) and $|G_{s}(f){|}^{2}$ are the same functions as those of the DMCA. The DMA scaling exponent α is estimated as the slope of a log-log plot of F(s) versus s. Using equation (3.2), we can analytically derive the scaling relations, α=1−γ/2 when the auto-correlation obeys $C(k)∼k^{-\gamma }$ (0<γ<1) and α=(β+1)/2 when the power spectral density obeys $S(f)∼f^{-\beta }$ (−1<β<2m+3) [22]. In this analysis, 0.5<α<1 indicates a long-range correlation, whereas 0<α<0.5 represents a long-range anti-correlation. When C(k) is the unit impulse function or decays exponentially to zero, the scaling behaviour results asymptotically in α=0.5.

In our long-range cross-correlation analysis, the second-order DMCA was used because the consistencies of the second- and fourth-order DMCA results were confirmed for our datasets (see §5(a)). $F_{12}^{2}(s,\kappa )$ and $\rho_{12}(s,\kappa )$ were estimated for each pair of time series, where the value of κ was determined based on the peak position in $|K_{12}(k)|$ (equation (2.19)). If no dominant peaks of $|K_{12}(k)|$ appeared, κ was set to be zero.

In this study, the group mean of $F_{12}(s,\kappa )$ at each s was calculated using the group mean of $F_{12}^{2}(s,\kappa )$. When $\rho_{12}(s,\kappa )$ consistently exhibits non-zero values (positive or negative) over the scaling range, it suggests that a common noise source exists. The long-range cross-correlation of the common noise source is characterized by the scale dependence of $F_{12}(s,\kappa )$. Using the scaling exponent α of $F_{12}(s,\kappa )∼s^{\alpha }$, we can characterize the long-range correlated behaviour of the common noise source. The interpretation of α is the same as that of univariate DMA.

In the original DMA and DMCA, the time scale s has been expressed by the number of data points. In this study, when we analyse a time series having a sampling frequency $f_{\text{samp}}$, the time scale is expressed by $\tilde{s}/f_{\text{samp}}$ in unit of second instead of the number of data points. As shown in table 1, the relation between s and $\tilde{s}$ depends on the order of DMA and DMCA. For instance, we use $\tilde{s}=s/1.93$ in the case of second-order DMA and DMCA.

#### (ii) Coherence analysis

We estimated the squared coherence [32] as a conventional approach for the cross-correlation analysis. The squared coherence of bivariate time series $\left\{(x^{\left(1\right)}[i],x^{\left(2\right)}[i])\right\}$ is defined as

3.3 $$|\Gamma_{12}(f){|}^{2}=\frac{|\left\langle S_{12}(f)\right\rangle {|}^{2}}{\left\langle S_{1}(f)\right\rangle \left\langle S_{2}(f)\right\rangle },$$

where $S_{12}(f)$ is the cross power spectrum between $x^{\left(1\right)}[i]$ and $x^{\left(2\right)}[i]$, $S_{1}(f)$ and $S_{2}(f)$ are the power spectra of $x^{\left(1\right)}[i]$ and $x^{\left(2\right)}[i]$, respectively, and ⟨ ⋅ ⟩ denotes Welch’s overlapped-segment averaging estimator for each spectrum. The squared coherence provides a measure of the degree of cross-correlation between the frequency components of $x^{\left(1\right)}[i]$ and $x^{\left(2\right)}[i]$.

#### (iii) Surrogate time series

We generated sets of surrogate time series without any cross-correlation and estimated the 95% confidence intervals for an uncorrelated case to determine the statistical significance of the observed cross-correlation between two time series. The surrogate time series were generated using an amplitude adjusted Fourier transform (AAFT) algorithm [33]. We initially generated a stochastic time series with the same power spectrum as the original time series using a Fourier phase randomization. The original time-series values were then sorted based on the order of the phase-randomized time series. We produced 100 surrogate time series with the AAFT algorithm that had the same distribution and a similar power spectrum as the original series, but no cross-correlation with the other time series. We estimated the 95% confidence intervals of each mean based on t-statistics.

#### (iv) Conventional HRV parameters

To provide the basic characteristics of HRV,the following HRV parameters were calculated: mean RR intervals (mean NN), standard deviation (s.d.) of RR intervals (SDNN), the variances corresponding to very low frequency (VLF; 0–0.04 Hz), low frequency (LF; 0.04–0.15 Hz), and high frequency (HF; 0.15–0.40 Hz) bands, and LF/HF ratio, all of which were proposed by the Task Force of the European Society of Cardiology and the North American Society of Pacing and Electrophysiology [1]. The variances of these frequency components were transformed to natural logarithmic values.

### (b) Data

We analyse two datasets obtained from healthy human subjects at rest. The first dataset consists of breathing airflow signals and HRV measurements. Electrocardiography (ECG) and nose-mouth airflow signals were simultaneously recorded in eight male subjects (22.4±0.9 yr (mean±s.d.)). Each subject was seated in a relaxed position for the three recordings (30 min each). Data were recorded with PowerLab and LabChart Pro software (AD Instruments) at a sampling frequency of 1000 Hz. Breathing airflow measurements were recorded with a thermistor flow sensor (NIHON SANTEKU Co., Ltd) attached to the subject’s nose and mouth. RR interval (RRI) time series derived from the ECG recordings were linearly interpolated and resampled at 2 Hz. The airflow signals were also resampled at 2 Hz. In addition, inter-breath intervals (IBI) were extracted by detecting the peaks and valleys in the airflow signals, linearly interpolated, and resampled at 2 Hz.

The second dataset consists of beat-to-beat systolic BP (SBP), diastolic BP (DBP), and RRI. ECG and continuous BP were simultaneously recorded at a sampling frequency of 1000 Hz in four male subjects (22.3±1.0 yr). A tonometric device (JENTOW7700, Colin Electronics, Japan) was used to measure continuous BP from the right wrist. To synchronize ECG and continuous BP, both signals were connected to PowerLab and LabChart Pro software (AD Instruments). Data were recorded for 20 min after resting for 5 min in a supine position on a bed. Beat-to-beat SBP and DBP were extracted from the peaks and valleys in the continuous BP signal. The RRI, SBP and DBP time series were then resampled at 2 Hz.

All erroneous peak detections in RRI, IBI, SBP and DBP time series were manually corrected based on the visual inspection of the signals.

## 4. Results

The baseline characteristics of conventional HRV parameters in each dataset are shown in table 2. In the cardiorespiratory dataset, mean IBI was 3.8±0.5 s (mean±SD). In the cardiovascular dataset, mean SBP and DBP were 108.6±7.2 mmHg and 50.9±2.4 mmHg, respectively.

**Table 2.** Baseline characteristics of HRV parameters of each dataset.

	cardiorespiratory data	cardiovascular data
	(mean ± s.d.)	(mean ± s.d.)
mean NN (ms)	.868±66	1017±151
SDNN (ms)	69.5±26.2	85.8±13.2
ln HF (${\text{ln ms}}^{2}$)	6.25±0.89	6.96±0.83
ln LF (${\text{ln ms}}^{2}$)	7.15±0.89	7.55±0.37
ln VLF (${\text{ln ms}}^{2}$)	7.71±0.59	7.92±0.57
LF/HF	2.77±1.04	2.66±2.32

**Figure 1.**
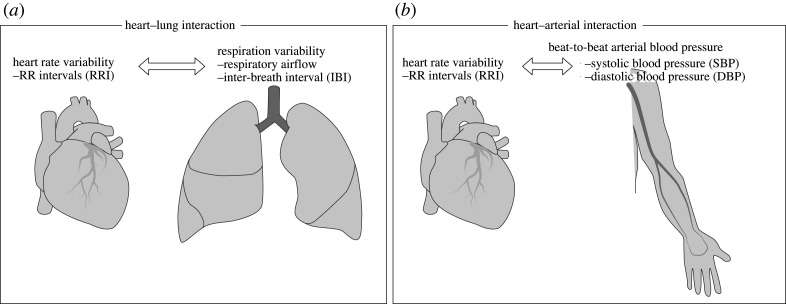
Cardio-respiratory (*a*) and cardiovascular and (*b*) datasets analysed in this study.

**Figure 2.**
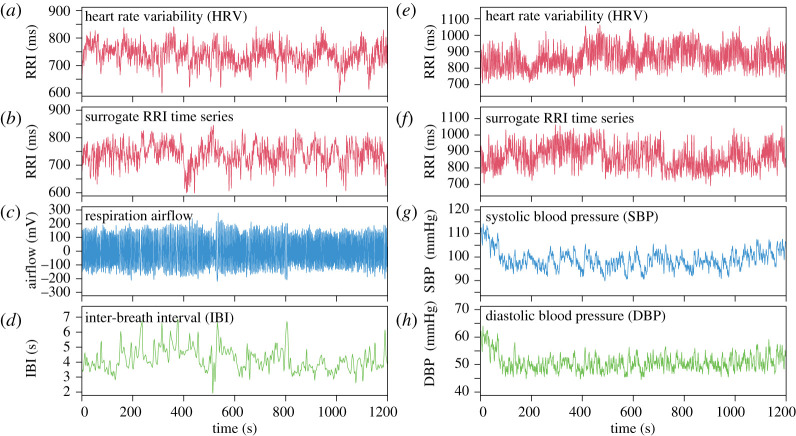
(Left) Representative cardiorespiratory time series: (*a*) RR interval (RRI), (*b*) surrogate RRI, (*c*) respiration airflow,(*d*) inter-breath interval (IBI). (Right) Representative cardiovascular time series: (*e*) RRI; (*f*) surrogate RRI, (*g*) Beat-to-beat systolic blood pressure (SBP), (*h*) beat-to-beat diastolic blood pressure (DBP). (Online version in colour.)

**Figure 3.**
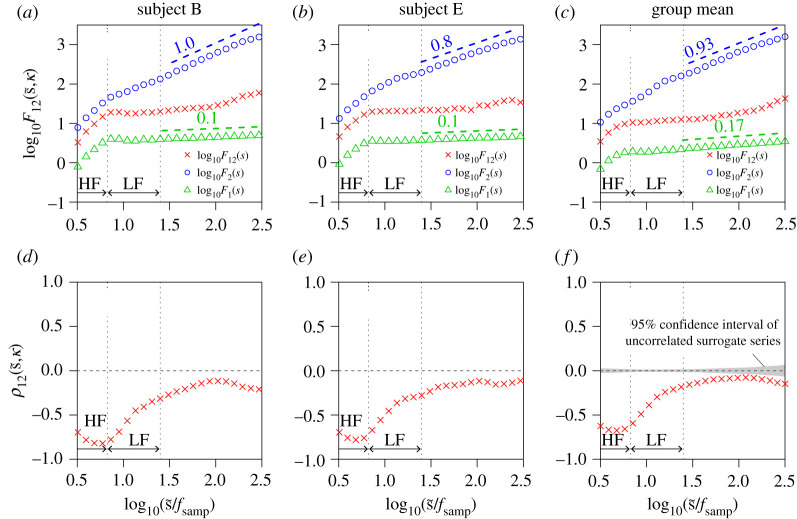
Long-range cross-correlation analysis for RRI and respiration airflow. (*a*–*c*) Univariate DMA results of RRI (circle) and respiration airflow (triangle) and bivariate DMCA results between RRI and respiration airflow (cross symbol). (*d*–*f*) Multi-scale cross-correlation coefficients between RRI and respiration airflow (cross symbol). The left and centre panels each show representative results (means of three recordings) for an individual subject. The rightmost panels show the groups means for all subjects. In the bivariate analysis, we set the lag as κ=0. Scaling exponents α were estimated in the range of $1.46\leq \log_{10}(\tilde{s}/f_{\text{samp}})\leq 2.42$ (corresponding to 30 to 270 s). The estimated slope values are indicated in the figure. (Online version in colour.)

**Figure 4.**
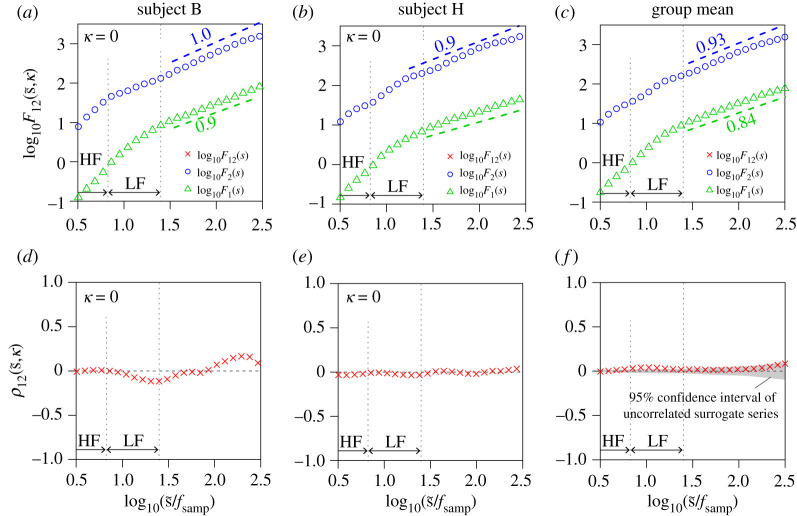
Long-range cross-correlation analysis for RRI (circle) and IBI (triangle). Other details are the same as figure 3. (Online version in colour.)

**Figure 5.**
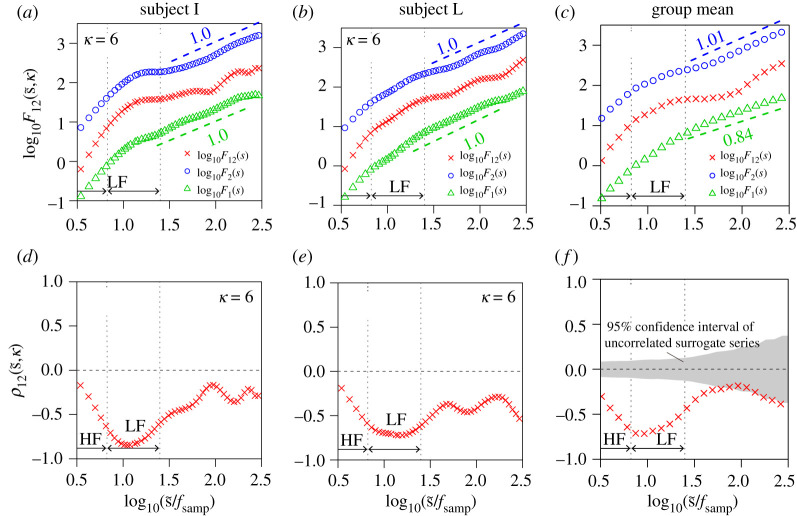
Long-range cross-correlation analysis for RRI and SBP. (*a*–*c*) Univariate DMA results of RRI (circle) and SBP (triangle) and bivariate DMCA results between RRI and SBP (cross symbol). (*d*–*f*) Multi-scale cross-correlation coefficients between RRI and SBP (cross symbol). The left and centre panels each show representative results (means of three recordings) for an individual subject. The rightmost panels show means for all subjects. In the bivariate analysis, we set the lag as κ=5.5±0.9 (SBP was 2.8 ± 0.4 s behind RRI). Scaling exponents α were estimated in the range of $1.46\leq \log_{10}(\tilde{s}/f_{\text{samp}})\leq 2.42$ (corresponding to 30 to 270 s). The estimated slopes are indicated in the figure. (Online version in colour.)

**Figure 6.**
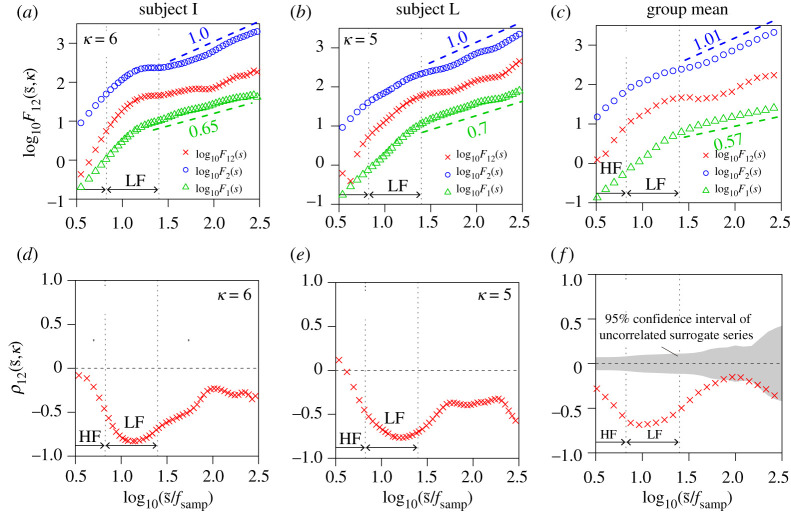
Long-range cross-correlation analysis for RRI (circle) and DBP (triangle). In the bivariate analysis, we set the lag as κ=4.3±1.3 (DBP was 2.1±0.7 s behind RRI). Other details are the same as figure 5. (Online version in colour.)

We evaluated long-range correlations between (i) respiration (airflow and IBI) and RRI and (ii) BP (beat-to-beat SBP and DBP) and HRV (figure 1). Representative time series, including the surrogate time series of RRI, are shown in figure 2. The results of the auto- and cross-correlation analyses using DMA and DMCA, respectively, are shown in figures 3 and 4 for the respiration-HRV interactions and in figures 5 and 6 for the BP-HRV interactions. In each figure, the panels in the left two columns show representative results for individual subjects, and the panels in the rightmost column show the group means for all subjects. The individual subject ID corresponds to the file name provided as electronic supplementary material.

### (a) Respiration and heart rate variability

The mean of the univariate DMA scaling exponents for RRI was $\alpha_{\text{RRI}}=0.93\pm 0.02$ (circles in figure 3*c*). Note that $\alpha_{\text{RRI}}=1$ corresponds to the 1/f fluctuation when β=1. The mean of the univariate DMA scaling exponents for respiration airflow was $\alpha_{\text{airflow}}=0.17\pm 0.02$ (triangles in figure 3*c*). Theoretically, if the respiration airflow exhibits a sinusoidal-like oscillation, the scaling exponent approaches zero asymptotically. However, the scaling exponent $\alpha_{\text{airflow}}$ appeared to be slightly greater than zero, which indicated a long-range anti-correlation.

We set the lag as κ=0 in the bivariate DMCA analysis because the primary peaks of $K_{12}(k)$ were observed around k=0 for respiration airflow and not clearly observed for IBI. The relation between the respiration airflow and RRI showed a significant cross-correlation at approximately 4 s in the HF band (figure 3*d*–*f*). The bivariate DMCA result (figure 3*c*) suggested that the RRI was weakly affected by the fluctuations associated with the respiration airflow in a long-range anti-correlated manner. The common factor between the respiration airflow and RRI behaved like the respiration airflow.

The mean of the univariate DMA scaling exponents for IBI was $\alpha_{\text{IBI}}=0.84\pm 0.01$ (circles in figure 4*c*). Although RRI and IBI exhibited long-range correlation, no common factor was observed. RRI and IBI were not cross-correlated over the entire time scale (figure 4*d*–*f*). Therefore, $F_{12}(s,\kappa )$ was not available (not depicted in figure 4*a*–*c*).

### (b) Blood pressure and heart rate variability

The means of the univariate DMA scaling exponents for RRI and SBP were $\alpha_{\text{RRI}}=1.01\pm 0.03$ and $\alpha_{\text{SBP}}=0.84\pm 0.02$, respectively (circles and triangles in figure 5*c*). Although the bivariate DMCA results showed an increasing trend in the double logarithmic plot of $F_{12}$, a scaling behaviour was not evident. Therefore, the estimated slope values are not shown.

The estimated lags between SBP and RRI were distributed around κ=6 (SBP is 3 s behind RRI). The relation between RRI and SBP revealed a significant cross-correlation around 10 s in the LF band (figure 5*d*–*f*). The results of each subject consistently suggested that a long-range cross-correlated factor between RRI and SBP existed. However, its correlation was weak, and the scaling law was not evident. The comparison with the surrogate RRI time series reveals that the significance of our observations was marginal (figure 5*f*).

The mean of the univariate DMA scaling exponents for DBP is $\alpha_{\text{DBP}}=0.57\pm 0.02$ (triangles in figure 6*c*), which was smaller than that of SBP and close to 0.5. Although the bivariate DMCA results exhibited an increasing trend in the double logarithmic plot of $F_{12}$, a scaling behaviour was not evident (like the relation between RRI and SBP). Therefore, the estimated slope values are not shown.

The existence of a long-range cross-correlated factor between RRI and DBP was suggested (as it is between RRI and SBP), although its significance compared with the surrogate RRI time series was less than that of RRI and SBP.

### (c) Coherence analysis results

**Figure 7.**
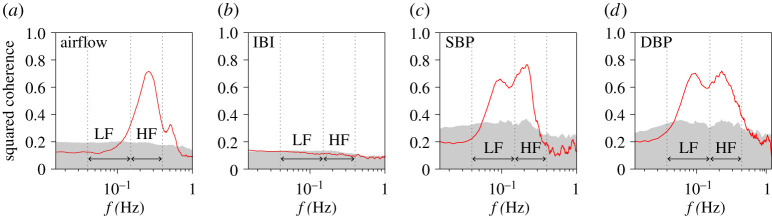
Coherence analysis results. Mean coherence with RRI over all subjects is described by red solid lines. (*a*) Respiration airflow (N=8), (*b*) IBI (N=8), (*c*) SBP (N=4), (*d*) DBP (N=4). The grey shaded areas show the 95% confidence intervals for the uncorrelated surrogate time series. (Online version in colour.)

The group means of the estimated squared coherence are shown in figure 7. The significantly cross-correlated fluctuations between respiration airflow and RRI were observed primarily in the HF band (figure 7*a*). Uncorrelated fluctuations between IBI and RRI were observed across all frequencies (figure 7*b*) and were consistent with the bivariate DMCA result (figure 4*f*). The significantly cross-correlated fluctuations between SBP and RRI (figure 7*c*) and between DBP and RRI (figure 7*d*) were observed in the HF and LF bands. In both cases, the peak frequencies of the squared coherence in the LF band were consistent with the bivariate DMCA results (figure 5*f* and 6*f*). In all cases shown in figure 7, no significant cross-correlations were observed in the very low-frequency band (f<0.04 Hz).

## 5. Discussion

### (a) Methodological notes on detrending moving-average cross-correlation analysis

An important scientific contribution of our study is the establishment of a methodological framework for long-range cross-correlation analyses using DMCA. The following procedures are required to achieve a reliable and valid estimate of the long-range cross-correlation quantified by $F_{12}(s,\kappa )$: (i) Time-scale correction in the higher-order DMCAs; (ii) consistency checks for the different order DMCAs; (iii) estimation of the time lag between two time series. These points have not been explicitly considered in previous studies using DMCA.

**Figure 8.**
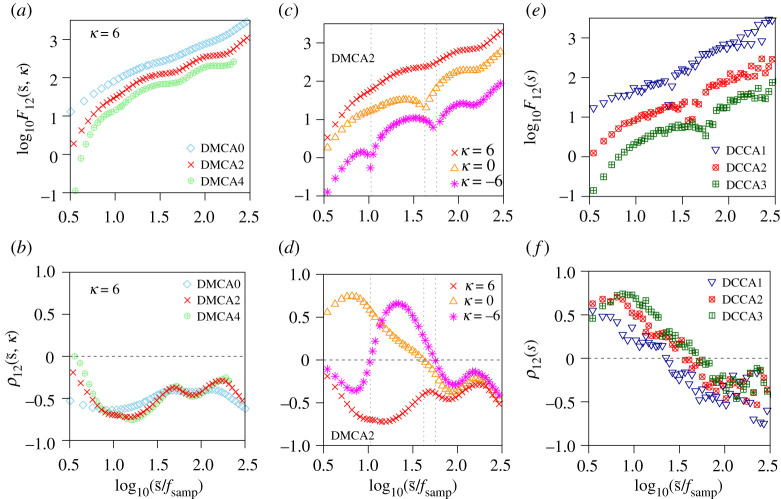
Comparison of bivariate analyses using different methods and parameters. RRI and SBP time series of subject L shown in figure 5*b*,*e* were analysed. (*a*,*b*) Results of zeroth-, second- and fourth-order DMCAs (denoted as DMCA0, DMCA2 and DMCA4, respectively) with κ=6. (*c*,*d*) Second-order DMCA results with κ=6, 0, −6. (*e*,*f*) Results of first-, second- and third-order DCCAs (denoted as DCCA1, DCCA2 and DCCA3, respectively) with κ=0. (Online version in colour.)

We illustrate the importance of the points mentioned above in figure 8, which shows bivariate DMCA and DCCA analyses of a single subject’s data consisting of RRI and SBP time series. The results obtained from the zeroth-, second- and fourth-order DMCAs (denoted as DMCA0, DMCA2 and DMCA4, respectively) with κ=6 are shown in figure 8*a*,*b*. Note that m-th order DMCA is equivalent to (m+1)-th order DMCA, where m is an even number. The DMCA2 and DMCA4 results of $\rho_{12}$ show the consistency for $\tilde{s}/f_{\text{samp}}>0.7$. This consistency indicates that the adverse effect induced by the non-stationary trend is sufficiently attenuated by using the second-order detrending. The corrected time scale $\tilde{s}$ makes it possible to confirm the consistency of the peak and valley positions in $F_{12}(s,\kappa )$ and $\rho_{12}(s,\kappa )$.

The second-order DMCA results with different lags κ=6,0,−6 are shown in figure 8*c*,*d*. In this case, $|K_{12}|$ (equation (2.19)) showed the highest peak at κ=6. Therefore, we used κ=6 in the analysis in the previous section. The scale dependence of $F_{12}(s,\kappa )$ and $\rho_{12}(s,\kappa )$ changes dramatically depending on κ. When κ=0 and κ=−6, the signs of $\rho_{12}(s,\kappa )$ change depending on the scale. $F_{12}(s,\kappa )$ exhibits sudden drops (vertical dotted lines in figure 8*c*,*d*) that correspond to the sign changes of $\rho_{12}(s,\kappa )$. Therefore, the $F_{12}(s,\kappa )$ does not provide any meaningful information without the appropriate lag.

We analysed the same data using DCCA (for methodological details see [27]) to compare DMCA and DCCA (figure 8*e*,*f*). In the original DCCA, the corrected scales and the lag estimation are not introduced. Therefore, the DCCA results are very different from our DMCA results. Moreover, the detrending operation causes the DCCA results to exhibit non-smooth behaviours. In the DCCA, non-stationary trends included in a time series are eliminated using a piecewise least-squares polynomial fitting. This procedure acts as nonlinear filtering and induces high-frequency fluctuations in the detrended time series [34]. Therefore, such high-frequency fluctuations cause non-smoothness in the DCCA results.

### (b) Cardio-respiratory and cardiovascular interactions

An oscillatory component observed in the HF range of HRV is modulated by respiration through a process known as the respiratory sinus arrhythmia (RSA) [35]. RSA is modulated by inhibition of cardiac parasympathetic activity during inspiration and return of the parasympathetic activity during expiration. To date, cardiorespiratory interactions have been studied mainly focusing on the oscillation component associated with RSA. Using conventional linear analysis methods such as coherence analysis, the significant interactions between respiration airflow and RRI were observed only in the HF range (figure 7*a*). By contrast, in our analysis using DMCA, the significant interactions between respiration airflow and RRI were observed in wider time scales including HF and LF bands (figure 3*f*). Therefore, our approach could provide a new and important insight into nonlinear interactions in the cardiorespiratory system. Because no correlations were observed between IBI and RRI (figure 4*f*), our findings suggest that the cardiorespiratory interactions are mainly modulated by the respiratory amplitude but not by the respiratory rate. To date, complex cardiorespiratory interactions been modelled and analysed using approaches in statistical and nonlinear physics [36,37]. Our approach could facilitate to understand the cardiorespiratory interactions.

The respiration variability described by IBI exhibits long-range auto-correlations. Although long-range correlations in IBI have been reported [38], the interaction between IBI and RRI has not been studied from the perspective of long-range cross-correlation. Our analysis revealed that there was no cross-correlated factor in IBI-RRI interactions. This finding did not help us understand the origin of the long-range auto-correlations of RRI. However, an uncorrelated relation between IBI and RRI suggests that respiration variability may have biomedical significance and applications independent of the well-established features of HRV.

Our results (figures 5 and 6) showed that SBP exhibits long-range auto-correlations and that SBP and RRI share a common long-range correlated factor. Strong negative correlations observed in the LF range (figure 5*f*) would be associated with Mayer wave oscillation. However, the common factor observed in the longer time scales (greater than 25 s) corresponding to the very low frequency range (less than 0.04 Hz) is a random fluctuation, because smooth sinusoidal waves are eliminated by the detrending procedure of higher-order DMCA. The estimated lags between RRI and BP indicates that RRI precedes BP. Therefore, the primary source of a long-range cross-correlation should be RRI. Previous studies have demonstrated that the Starling law and diastolic runoff could explain the causal direction of the interaction from HRV to SBP in the LF band [39]. Therefore, the long-range cross-correlation between HRV and SBP may have the same mechanical origin. Previous studies have proposed that the long-range auto-correlated BP variability has medical significance [40–42]. Therefore, the long-range cross-correlation between BP and HRV may represent a new medical bio-marker [43].

Definitely, our findings are still preliminary and require further validation. We tested data only from healthy young male adult subjects under resting condition. Thus, characteristics of the long-range cross-correlation, such as body posture, age and sex dependence, are not fully evaluated. To understand the characteristics and mechanism of cardiorespiratory and cardiovascular interactions, a more extensive and more systematic study is required.

## 6. Conclusion

We illustrated the practical usage of higher-order DMCA with a Savitzky–Golay filter and demonstrated its ability to detect long-range cross-correlations. The following three points are essential to achieve a reliable characterization of long-range cross-correlations using DMA and DMCA: (i) time-scale corrections in higher-order DMCAs, (ii) consistency checks of different order DMCAs and (iii) time-lag estimations.

Our approach provided new insights into cardiorespiratory and cardiovascular interactions. IBI and RRI showed long-range auto-correlations in cardiorespiratory interactions but did not share any common factor in the analysed scale. This finding suggests that the cardiorespiratory interactions are mainly modulated by the respiratory amplitude but not by the respiratory rate. In cardiovascular interactions, SBP and RRI showed long-range auto-correlations. In addition, they shared a common factor in the analysed scale, although the scaling law of the cross-correlation was not evident.

Long-range correlations are ubiquitous in real-world time series [44]. Hence, Our approach is well-suited to applications in multivariate time-series analysis.

## Data accessibility

Data are available as electronic supplementary material.
